# Positive Emotion Regulations Among English as a Foreign Language Teachers During COVID-19

**DOI:** 10.3389/fpsyg.2021.807541

**Published:** 2021-12-13

**Authors:** Hongdan Zhao

**Affiliations:** College of Education, Arts and Sciences, Lyceum of the Philippines University, Batangas, Philippines

**Keywords:** positive psychology, positive emotion, emotion regulation, COVID-19, EFL teacher

## Abstract

As the cores of education, teachers’ emotions have a critical place in academia. However, the power of EFL (English as a foreign language) teachers’ positive emotions and their regulation in online mode of instruction have been ignored by scholars. With the rapid shift of education from face-to-face to remote/electronic delivery, many challenges and emotional problems emerged among teachers and learners worldwide. This entailed the necessity of considering and planning for emotional regulation to generate positive outcomes. To provide a roadmap for this line of research, the present mini-review article presented the theoretical and empirical underpinnings of emotion regulation, its origins and definitions, as well as outcomes for second/foreign language education. The study also presents some implications for EFL teachers, teacher trainers, and avid scholars of this area of research pinpointing the current yawning gaps.

## Introduction

Emotions and inner feelings now play a significant role in education, in general, and in language teaching and learning, in particular (Mercer, 2020; Sikma, 2021). Proper awareness and management of such emotions largely determine academic success and practice (Gregersen and MacIntyre, 2021). This turn of focus toward emotions emanated from positive psychology (PP) and the affective turn which highlighted the role of positive feelings and states in one’s success and achievement in academic arena (Zhang and Zhang, 2020; Wang and Derakhshan, 2021). The result of this prominence given to positive emotions in second/foreign language education was a growing surge of scholarly interest stressing various constructs and dimensions of this line of research (e.g., Seligman, 2011; Li and Yang, 2021; Sun, 2021; Xie and Derakhshan, 2021). It is widely embraced that EFL teachers, as the cores of education, face a multitude of emotions and stressors during their profession as one of the most demanding jobs ever (Benevene et al., 2020). They have to deal with instructional, cultural, emotional, and mental challenges and disparities at the same time. This necessitates a strong emotional-regulation system in which the teachers control and manage their experienced emotions before, during, and after their emergence (Wang and Ye, 2021). For EFL teachers, this capability to regulate emotions is a pivotal socio-emotional skill that brings about resilience and flexibility in facing adverse situations common in teaching (Cam, 2021; Wijaya, 2021). In other words, it is a mechanism which can improve, maintain, and reduce the timing, duration, intensity, and construction process of both negative and positive emotions (Koole, 2009).

Aside from pedagogical knowledge and expertise, EFL teachers need psycho-emotional knowledge as well. A teacher may be able to teach English efficiently but if he/she does not know how to manage and express his/her emotions, the teaching and learning processes may go astray. Consequently, EFL teachers have to use different strategies to suppress negative emotions and, contrarily, augment and increase positive feelings which, in turn, determine teaching effectiveness and students’ learning and engagement (Wang and Ye, 2021). Before the rise of PP, most studies on teachers’ emotions were limited to negative emotions and how to modify them (Cregg, 2017). However, in the past couple of years due to the precious works of some scholars of this school of psychology, teachers’ positive emotions have caught a growing attention among researchers (e.g., MacIntyre and Mercer, 2014; Mercer, 2020; Wang et al., 2021; Xie and Derakhshan, 2021; Zhao and Li, 2021, to name a few). These studies led to numerous positive outcomes in L2 education including improved resilience, self-esteem, self-efficacy, motivation, interpersonal skills, confidence, success, well-being, academic achievement and many more. Nevertheless, with the outbreak of COVID-19 that forced all educators to resort to a new mode of instruction and keep up with physical distancing protocol, the criticality and power of emotions multiplied. Yet, the existing studies on emotions and the regulation of such feelings in online milieus are confined to students’ negative emotions like stress, boredom, and disengagement (Derakhshan, 2021; Derakhshan et al., 2021; Devkota, 2021). Against this lacuna, the present article aimed to review the theoretical and empirical underpinnings of teachers’ positive emotion regulation during the outbreak and offer some recommendations for further inquiry.

## Emotion and Related Theories

### Emotions

L2 teaching is a job full of psycho-emotional variables that influence each and every stage of pedagogy and learning (Li, 2021). The first stepping stones of this shift of attention toward the role of emotions in education were laid by humanistic psychology and positive psychology (PP, hereafter). In contrast to constructivism that turned a blind eye on emotions, these two paradigms capitalized on the role of emotions, especially positive emotions, in generating happiness, satisfaction, development, and a sense of life flourish (MacIntyre et al., 2019). While it approves negative emotions, PP deals with the positive sides of life and argues that positive emotions can solve negativities and adversities (Seligman, 2011; Zhang and Zhang, 2020). Based on humanism and PP, EFL students and teachers develop and demonstrate a high performance only if their inner emotions, personal characteristics, and styles are taken into account in an academic context. Certifying these claims, research substantiated that constructs related to EFL teachers’ positive emotions comprising joy, love, resilience, mood, buoyancy, outlook, optimism, engagement, interpersonal skills, hope, passion, and enjoyment can develop their classroom performance and solve many educational problems (Dewaele et al., 2019; Wang and Derakhshan, 2021).

Moreover, this pedagogy of affect and emotion can improve EFL teachers’ positive interpersonal relations, well-being, communication abilities (e.g., credibility, clarity, conformation, immediacy) and establish a peaceful educational climate that ultimately causes students’ academic achievement (Gibson, 2011; Ainsworth and Bell, 2020). The underlying purpose is to identify, arouse, and improve teachers’ positive emotions and the way they can be regulated to shape a psycho-pedagogy trend (Williamson, 2016).

### Emotion Regulation

The notion of emotion regulation refers to one’s ability to control, handle, modify, and regulate the perception and expression of emotions caused by internal and external factors (Wijaya, 2021). It is a process by which people make attempts to affect their encountering emotional experiences to accomplish personal ends (Colombo et al., 2021). It is regarded as a social-emotional construct that focuses on both the maintenance and improvement of emotions as well as their inhibition and control (Akbari et al., 2017). The concept can be automatic/unconscious or conscious depending on the emotional trigger/stimuli (Gross, 2002). From a different stance, emotion regulation can be seen as an individual difference trait which is not sensitive to time and context and is somewhat stable (Gross and John, 2003), yet it varies across age groups (Riediger and Luong, 2015; Röcke et al., 2018).

### Teachers’ Emotion Regulation

Teaching as a profession is highly entangled with emotions and intra-psychological factors and variables. Hence, academic success and performance of teachers largely hinges upon their ability to perceive and deal with such emotions. Based on this, it can be argued that teachers’ emotion regulation refers to their capacity to manage and sustain classroom emotional experiences (Fried, 2011; Wang and Ye, 2021). This management can be perception, expression, modification, maintenance, and development of emotional encounters by the teachers. In the context of L2 education, which is full of adversities and challenges, the importance of this mental mechanism increases radically. This ability can be achieved by employing different strategies depending on the emerging timing of the emotion. This means that regulation strategies can be *response-focused* or *antecedent-focused* (Greenier et al., 2021). Moreover, EFL teachers may *up-regulate* their emotions in order to improve teaching efficacy and deal with instructional tasks. Likewise, they may *down-regulate* their emotions to thwart negative impacts on learners’ classroom involvement, participation, performance, or motivation (Gong et al., 2013). In sum, teacher strategies in this domain can be classified into savoring strategies (beneficial) and dampening strategies (detrimental). The former refers to teachers’ strategies to increase positive emotional experiences, while the latter pertains to strategies utilized to reduce positive feeling (Bryant, 2003; Wood et al., 2003; Quoidbach et al., 2010; Wang and Guan, 2020).

Savoring strategies can be further divided into (1) *Behavioral Display* (expressing positive motions *via* non-verbal signals), (2) *Be Present* (purposely dragging attention toward the existing pleasing experience), (3) *Capitalizing* (sharing and rejoicing positive events with others), and (4) *Positive Mental Time Travel* (clearly recalling or expecting positive events). In a similar manner, dampening strategies can be categorized into (1) *Suppression* (curbing or concealing positive emotions because of shyness, fear, or modesty), (2) *Distraction* (involving in worries, thoughts, and activities unrelated to the existing positive event), (3) *Fault Finding* (lingering/focusing on the negative elements of a positive situation), and (4) *Negative Mental Time Travel* which includes negative reminiscence and negative anticipations of future penalties. By using these strategies, EFL teachers can bring about various academic outcomes (positive and negative).

## The Outcomes of Positive Emotion Regulations: Empirical Underpinnings

Tracing the short history of researching this domain, one can identify that scrutinizing teachers’ regulation of emotions, in general, and positive emotions, in particular contributes to different aspects of teaching. As research indicates, teachers’ emotion regulation, in the classroom, can increase teaching effectiveness, establish a caring rapport with pupils, provide an ideal teacher-image for learners, increase well-being, motivation, engagement, and improve classroom discipline (Sutton, 2010; Jiang et al., 2016; Greenier et al., 2021). Nevertheless, in applied linguistics and L2 contexts, emotion regulation and its’ positive outcomes has been studied by a few scholars (e.g., Ghanizadeh and Royaei, 2015; Akbari et al., 2017; Talbot and Mercer, 2018; Fathi and Derakhshan, 2019). As an instance, Ghanizadeh and Royaei (2015) studied emotion regulation and emotional labor strategies in relation to teacher burnout. In doing so, three valid questionnaires of “*Emotion Regulation Questionnaire* (ERQ),” “*Teacher Emotional Labor Strategy Scale* (TELSS),” and “*Maslach Burnout Inventory* (MBI)” were distributed among 153 EFL teachers. Analyzing participants’ answers to the aforementioned questionnaires, the researchers found a negative association between emotion regulation and emotional labor strategies and EFL teachers’ burnout. By the same token, in a pure qualitative inquiry, Akbari et al. (2017) also delved into the consequences of teachers’ emotion regulation in EFL classes. Interviewing 18 EFL teachers, the researchers found that various emotion regulation strategies such as reappraisal, attention direction, and teaching context adjustments that teachers commonly employ in classrooms may result in a range of positive academic outcomes. Further, Fathi and Derakhshan (2019) also examined 256 EFL teachers’ stress in relation to their self-efficacy and emotion-regulation tendencies. The results revealed that teacher self-efficacy and emotion regulation could significantly predict EFL teachers’ stress. Likewise, Greenier et al. (2021) also carried out a seminal study on 108 British and 255 Iranian English language teachers’ emotion regulation, psychological well-being, and work engagement. They aimed to examine the predictive power of teachers’ emotion regulation and well-being and the results of their study demonstrated that both variables significantly predicted work engagement. Additionally, in Japan, Littleton (2018) investigated four Japanese ESL teachers’ emotion regulation strategies through interviews and found that despite some differences, the participants followed a similar process to regulate emotions in the classroom and their responses had a recognizable pattern. This slowly growing body of research on emotion regulation in EFL contexts, particularly in relation to teachers, is surprising as emotions are “the heart of language learning and teaching”. Most of the existing studies on emotions in L2 language education have focused on negative emotions such as stress, anxiety, tension, disengagement, demotivation, burnout, hopelessness and the like, while the role of positive emotions’ regulation and their potentialities have been overlooked (Wang and Guan, 2020; Greenier et al., 2021). Furthermore, in the current era of COVID-19 pandemic, which brought about an abrupt shift in pedagogy and learning pushing and prompting e-teaching and e-learning through various online platforms, the role of emotions and their regulation is more critical than traditional modes of education. This abrupt shift, *per se*, caused many problems and challenges for both teachers and learners.

## Discussion

In this review article, it was argued that EFL teachers’ emotions play a momentous role in learning and teaching. However, many existing studies in this domain have been limited to negative emotions with online mode of delivery being ignored. Also, the study enumerated some positive outcomes associated to highlighting emotion regulation in L2 education. As a result, the study can be valuable for EFL teachers in that they can raise their awareness and use of proper emotion regulation strategies to improve or reduce specific feelings in the class to contribute to a better teaching. They can realize the importance of emotions in language education and think more deeply about their own inner states and their pupils and devise appropriate techniques to flourish them. The ideas proposed in this review are also beneficial for teacher educators in the sense that they can conduct training courses, seminars, webinars, conferences, and expert meetings for novice and experienced EFL teachers during the pandemic. Through practical teaching techniques, they can improve their knowledge and practice of positive emotion regulation as well. Additionally, the findings of this review could be illuminating for policy-makers as well. Given the importance of psycho-emotional factors in education, policy-makers are expected to consider these factors in their proposed curriculums to academic centers. Finally, the study has implications for EFL researchers in that they can conduct similar investigations in this domain and fill the existing gaps. As a case in point, they can run mixed-methods studies on teachers’ emotion regulation during the pandemic and provide a richer account of the issue. Moreover, as reviewed, many studies on emotions are yet confined to negative emotions, so future studies can be done on positive emotions of both teachers and learners. Cross-cultural explorations are also suggested to eager scholars to see if teachers coming from various cultures use identifiable or different emotion regulation strategies. Likewise, positive emotion regulation can be examined through correlational studies with other variables of PP (see Wang et al., 2021; Xie and Derakhshan, 2021). The intersection of positive interpersonal communication skills such as credibility, clarity, immediacy, stroke, and confirmation is also a possible line for future research. Finally, future researchers are recommended to run longitudinal studies on EFL teachers’ positive emotion regulation using qualitative tools to depict the developmental pathways of regulatory strategies.

## Author Contributions

The author confirms being the sole contributor of this work and has approved it for publication.

## Conflict of Interest

The author declares that the research was conducted in the absence of any commercial or financial relationships that could be construed as a potential conflict of interest.

## Publisher’s Note

All claims expressed in this article are solely those of the authors and do not necessarily represent those of their affiliated organizations, or those of the publisher, the editors and the reviewers. Any product that may be evaluated in this article, or claim that may be made by its manufacturer, is not guaranteed or endorsed by the publisher.
